# Case report of glomerular histiocytosis associated with non-crystalline IgG-kappa paraproteinemia

**DOI:** 10.1186/s12882-025-03986-8

**Published:** 2025-02-05

**Authors:** Aditya Kishore, Susanna A. McRae, David Telio, Monica C. Beaulieu

**Affiliations:** 1 Division of Nephrology, University of British Columbia, Vancouver, Canada; 2 Department of Pathology and Laboratory Medicine, Providence Health Care, St Paul’s Hospital, Vancouver, Canada; 3 Faculty of Healthy Sciences and Medicine, Bond University, Gold Coast, Australia; 4 Division of Medical Oncology, Burnaby Hospital, Fraser Health Authority, Burnaby, Canada

**Keywords:** Crystal storing histiocytosis, Glomerular histiocytosis, Monoclonal gammopathy of renal significance

## Abstract

**Background:**

Monoclonal gammopathy of renal significance (MGRS) represents a range of disease processes arising from monoclonal proteins depositing in the kidney. These deposits vary and can be broadly grouped as containing a substructure or being non-organised. Their clinical phenotype can include proteinuria, haematuria, kidney injury and tubulopathies resulting in electrolyte changes.

**Case presentation:**

Crystal storing histiocytosis (CSH) is a rare form of MGRS that typically deposits in the interstitium but rarely in the glomerulus to cause progressive kidney disease. We report a case of a male with known monoclonal protein and progressive proteinuria, whose biopsy showed glomerular histiocytosis with non-crystallizing IgG kappa inclusions.

**Conclusion:**

This case reviews an unusual case of a glomerular histiocytosis with non-crystallizing IgG kappa inclusions.

**Supplementary Information:**

The online version contains supplementary material available at 10.1186/s12882-025-03986-8.

## Introduction

Lymphoproliferative monoclonal disorders have been established to cause kidney disease via several mechanisms based on the structure they take and area in which they deposit.

Cast nephropathy represents a myeloma defining event warranting treatment. However, there are several other kidney disease processes from deposition of monoclonal protein representing a range of monoclonal gammopathies of renal significance (MGRS). These deposits can be broadly grouped based on those that have a substructure and those that are non-organized deposits. Crystal storing histiocytosis (CSH) is a rare form of MGRS that typically deposits in the interstitium but rarely in the glomerulus to cause renal disease. We report a case of a glomerular histiocytosis with non-crystallizing IgG kappa inclusions.

## Case report

The patient was a 69-year-old male with a background of hypertension and monoclonal gammopathy of undetermined significance (MGUS), referred to nephrology clinic for proteinuria. His creatinine at the time was 99 µmol/L, with a glomerular filtration rate (GFR) of 67mL/min. He had a 24-hour urine protein excretion of 1.2 g/day with concordant urine albumin creatinine ratio (uACR) and urine protein creatinine ratios (uPCR) and 3–10 red blood cells on urinalysis.

His MGUS was diagnosed in February 2019 based on peripheral blood serum protein electrophoresis showing monoclonal bands of 15.2 g/L IgG kappa. He had a slightly elevated serum kappa to lambda ratio of 2.21 and urine protein electrophoresis with 3% IgG kappa. He had no associated hypercalcemia, renal dysfunction, anemia or bone involvement and an unremarkable skeletal survey, but at that stage no confirmatory bone marrow biopsy. At that point in 2019, he had a normal albumin of 39 g/L, some mild peripheral oedema and well controlled hypertension on amlodipine 10 mg daily and ramipril 10 mg daily.

**Fig. 1:**
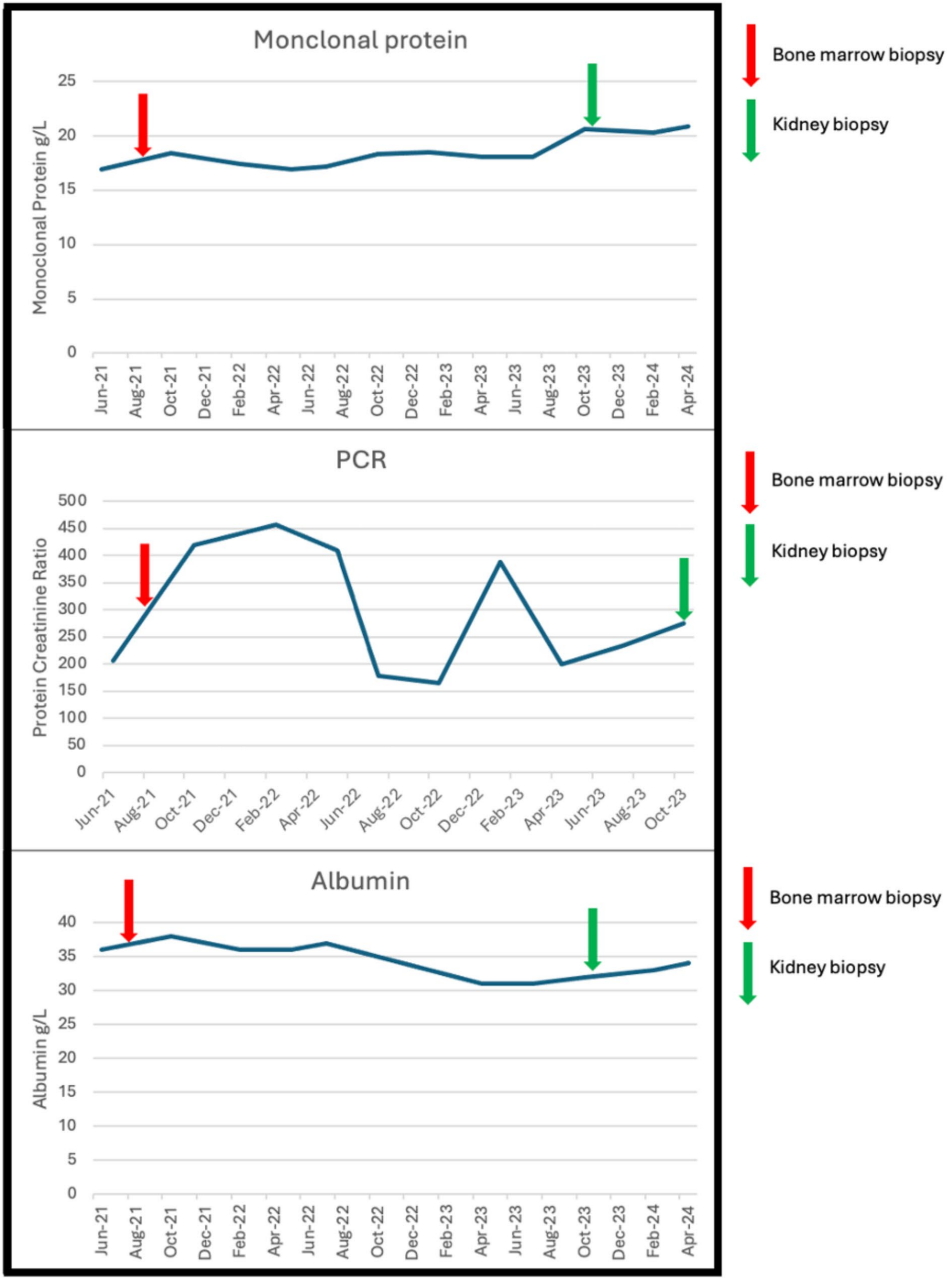
Serum monoclonal protein, urine protein creatinine Ratio (PCR) and serum albumin level over time

His IgG kappa monoclonal protein level progressed over two years to 18.4 g/L in August 2021 (Fig. 1) which resulted in a bone marrow biopsy showing less than 10% plasma cells although there was a large aggregate concerning for evolving plasma cell neoplasm. The Congo red stain was negative for amyloidosis. His renal function including proteinuria and serum creatinine and albumin remained stable. A kidney biopsy was recommended but at this point the patient wished to avoid invasive investigations including biopsy.

**Fig. 2:**
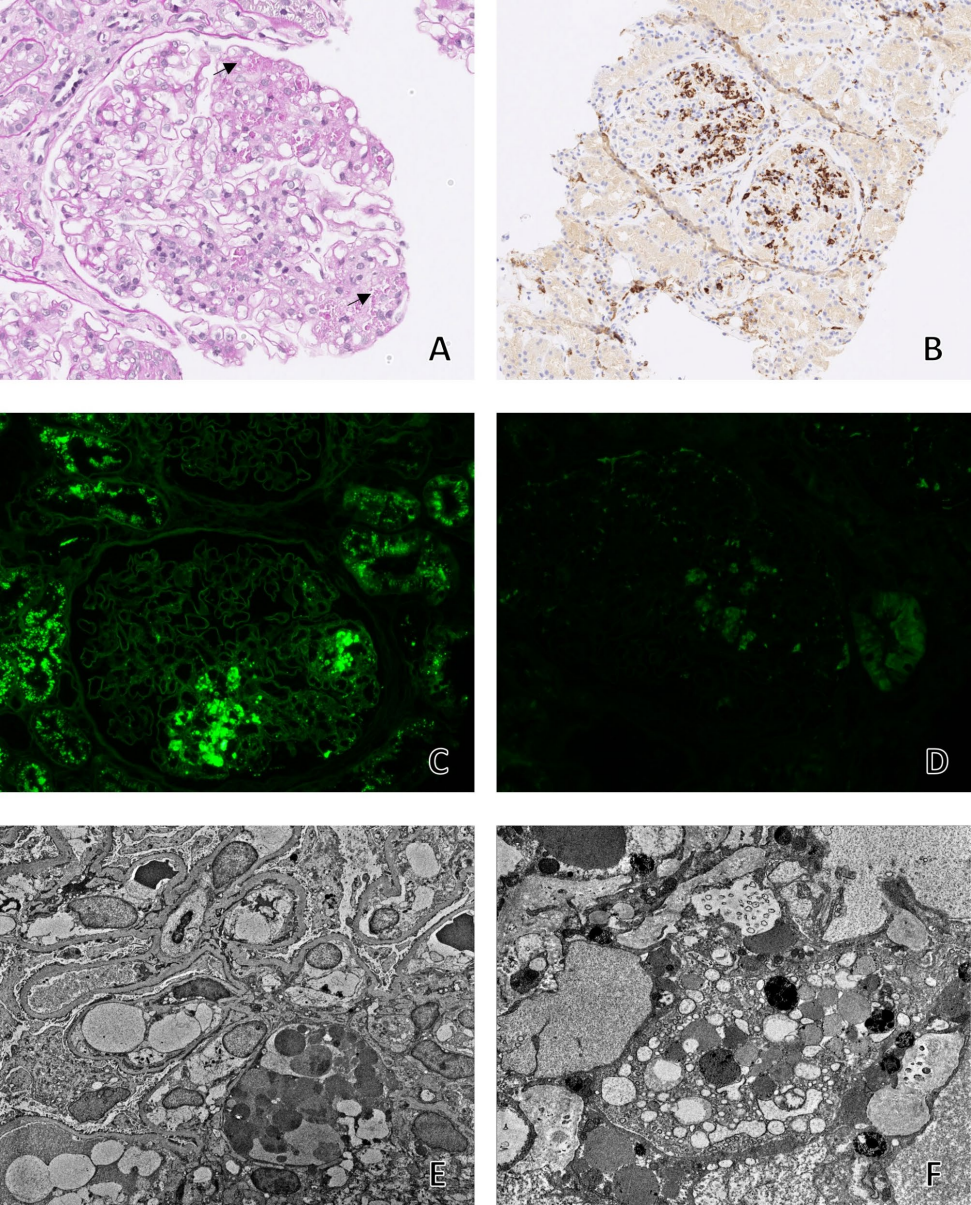
Kidney biopsy findings in histiocytic glomerulopathy withoutcrystals. Light microscopy shows segmental to global mesangial expansion by cellular component with prominent resorption droplets (**A**, PASx200),confirmed to be histiocytes by immunohistochemistry (**B**, CD163 × 100).Immunofluorescence, both direct on frozen tissue and performed on formalin fixed tissue with pronase digestion shows cytoplasmic staining within these histiocytes limited to IgG and kappa (**C**, kappax200; **D**, lambdax200). Ultrastructurally, the cytoplasm of these cells is distended with numerous electrondense granules, but no crystals (**E**, EMx2500; **F**, EMx12000)

He was continued on ramipril 10 mg, but he was intolerant of empagliflozin due to urinary frequency. He was monitored over the next two years with stable creatinine but increasing proteinuria and proceeded to renal biopsy in November 2023 (Fig. 2).

The renal biopsy showed three good fragments of renal tissue consisting of cortex, capsule and medulla with total of 36 glomeruli, 6% of which were globally sclerosed. Most glomeruli (23) showed segmental to global marked mesangial expansion by histiocytes staining positive for CD68 and CD163. The histiocytes contained prominent intracytoplasmic droplets, but no crystals. Close examination on multiple levels showed preservation of the peripheral capillaries, although segmental sclerosis with adhesions to Bowman’s capsule was noted in two glomeruli. There was no overt endocapillary hypercellularity, although rare glomerular basement membranes showed segmental remodeling. The mesangial segments without histiocytic involvement were generally unremarkable, with occasional mild hypercellularity. 5–10% interstitial fibrosis and tubular atrophy was seen. Interstitial histiocytes with prominent resorption droplets and/or crystals were not identified. No atypical casts or tubular intracytoplasmic crystals were seen. Congo red stain was negative.

On immunofluorescence there was focal and segmental staining within glomerular droplets for IgG (2+), C3 (1–2+), kappa (2+), and albumin (3+), with no staining along tubular basement membranes or in the interstitium. Arterioles stained for C3 only and tubular resorption droplets stained for kappa and albumin only. Immunofluorescence on paraffin embedded tissue following pronase digestion replicated these findings, and showed in better detail, the staining limited to the droplets. Apart from these discrete droplets, there was no staining within the mesangium, capillary walls, tubular basement membranes, blood vessels or interstitum. Tubular epithelial cells showed resorption droplets staining for IgG and kappa as well.

Electron microscopy showed a mesangium markedly expanded by mononuclear cells with numerous electron dense lysosomal granules of various sizes, but no crystals were identified. Tubular epithelial cells also contained multiple dense lysosomal granules but no crystals. Glomerular segments with histiocytic involvement showed some basement membrane remodeling with mesangial interpositioning, but the capillary loops remained patent. There were no significant mesangial, subendothelial, intramembranous, subepithelial or extraglomerular deposits.

## Discussion

There is a wide spectrum of monoclonal immunoglobulin-related disease. While overt myeloma has a clear indication for treatment, the timing and regimen of treatment for smoldering myeloma, monoclonal gammopathy of undetermined significance and various B cell lymphoproliferative disorders is less clearly defined. Light chain cast nephropathy is a myeloma defining event with acute kidney injury that warrants treatment. However, other monoclonal gammopathies of renal significance (MGRS) can present as slowly progressive kidney injury, proteinuria or hematuria and require biopsy to differentiate them from other common causes of kidney injury. Monoclonal deposition can be further divided by the presence of a substructure. A rare cause of MGRS monoclonal deposition with substructure is histiocytosis that contains crystalline light chains known as crystal storing histiocytosis. We report a rare case of progressive renal injury and proteinuria associated with a histiocytosis with non-crystalized IgG kappa inclusions.

Histiocytes themselves refer to either cells from tissue macrophage or dendritic cell lineage that accumulate in response to injury and inflammation [1]. Histiocytic migration into glomeruli can be seen in a number of pathological conditions including histiocytic glomerulopathy, thrombotic microangiopathy, lipoprotein glomerulopathy and lecithin-cholesterol acetyltransferase deficiency associated glomerulopathy [2]. In CSH, a histiocytic response is elicited in response to excess immunoglobulin typically from monoclonal proliferative disease. Here, histiocytes pinocytose paraproteins, in particular light chains, which leak out from damaged tubular cells [3, 4]. In typical CSH, histiocytes then degrade light chains into pseudo-pseudo-Gaucher cells and then undergo crystallization within histiocytic lysosomes. There is no clear heavy chain subtype that is more associated with the development of CSH, however, there is certainly a clear predominance of kappa light chain associated with CSH [5]. Lebeau et al. propose this may be due to poor kappa light chain solubility in acidic intralysosomal conditions [6]. They further suggest that alteration in amino acid substitutions promote hydrophobic residues which resist usual lysosomal proteolytic hydrolysis and promote crystallisation [5, 6]. 

Crystal storing histiocytosis (CSH) is characterized histologically by intracellular crystalline inclusions of monoclonal immunoglobulin deposits within lysosomes of histiocytes [7]. These inclusions can be found in any organ but uncommonly can be isolated to the kidney [8]. Renal CSH has been mostly described within the interstitium and rarely within the glomerular mesangium [9]. Shah et al. discussed the first case of glomerular CSH within the capillary loops [10]. Gupta et al. further reviewed cases of renal CSH involving the interstitium and glomerulus and found cases of glomerular CSH tended to have slower GFR decline with lower grade proteinuria [11]. All these cases involve crystalline substructure of various configurations including rhomboid, globular and needle shaped. Katsuma et al. report a case of MGRS with histiocytosis and non-crystalline kappa inclusions presenting with nephrotic syndrome and responding to myeloma treatment. The prognosis of renal histiocytosis in general is not clear due to its rarity, but mortality is linked with renal failure [12]. 

Our case not only contains glomerular histiocytic involvement without crystals, but also showed acute tubular injury with a kappa bias within tubular epithelial cells by immunofluorescence, raising the possibility of a non-crystalline form of light chain proximal tubulopathy (LCPT). Plasma cell dyscrasias with excess free light chain deposition exceed both the capacity of proximal tubule epithelial cells to resorb or effectively maintain lysosomal degradation. The excess free light chains can either crystalize within cytoplasm or cause increased atypical lysosomes without crystallization [13]. The overloaded lysosomes within tubular epithelial cells can cause direct acute tubular necrosis or impair tubular function leading to an acquired Fanconi syndrome [14]. Lan et al. note significant heterogeneity in free light chain toxicity and damage to tubular epithelial cells but do suggest that, similar to CSH, kappa light chains are more likely to cause crystallization than lambda light chains due to inherent chemical differences [13]. The non-specific electron microscopy findings of non-crystalline LCPT such as lysosomal abnormalities must be interpreted with caution as these findings could potentially represent a physiological trafficking of light chains [11]. 

Ungari et al. describe a case of concomitant CSH and non-crystalline LCPT presenting with predominant albuminuria [14]. However, Stokes et al.’s comparison of crystalline and non-crystalline LCPT cases, revealed that a majority of cases had proteinuria which was the result of light chain tubular protein. Due to the rarity of non-crystalline cases, they were not able to clearly draw any significant clinical or prognostic differences between crystalline and non-crystalline forms [15]. Kousios et al. review LCPT and suggest that non crystalline variants have a heterogenous histological presentation but often have slowly progressing CKD with proteinuria but no nephrotic syndrome. While there is no large study to confidently describe clear treatment outcomes for patients with MGRS LCPT, treatment directed against monoclonal disease does seem to improve kidney outcomes [16]. 

The presence of MGRS is grounds for treatment to suppress and eliminate the monoclonal protein causing direct kidney damage. Given the risk of further, potentially irreversible kidney injury we discussed treatment options for the paraproteinemia at length with the patient. Our preference would be to commence treatment with daratumumab, bortezomib and dexamethasone. Additionally, we advocated for a repeat bone marrow biopsy to rule out progression to multiple myeloma and help guide treatment with a daratumumab based regimen. In this case however, our patient was well-functioning, systemically well with a very slow decline in GFR and rise in proteinuria with no hypoalbuminemia or edematous complications, so he elected not to proceed with repeat bone marrow biosy or commence therapy for the monoclonal disease. He continues to have close outpatient follow up.

In summary, this is a rare case report of a glomerular histiocytosis associated with non-crystalline IgG kappa deposits. While the patient elected not to proceed with treatment till this point, he remains under close follow up for progression of disease.

## Electronic supplementary material

Below is the link to the electronic supplementary material.

Supplementary Material 1

Supplementary Material 2

Supplementary Material 3

Supplementary Material 4

Supplementary Material 5

Supplementary Material 6

## Data Availability

No datasets were generated or analysed during the current study.

## Abbreviations

CSH: Crystal storing histiocytosis
MGRS: Monoclonal gammopathies of renal significance
MGUS: Monoclonal gammopathy of undetermined significance
GFR: Glomerular filtration rate
uACR: Urine albumin creatinine ratio
uPCR: Urine protein creatinine ratios
LCPT: Light chain proximal tubulopathy
