# Supplementary figures and images for: Case report of glomerular histiocytosis associated with non-crystalline IgG-kappa paraproteinemia
Source: BMC Nephrol. 2025 Feb 5;26:56. doi: 10.1186/s12882-025-03986-8 (PMC11796192; doi:10.1186/s12882-025-03986-8)

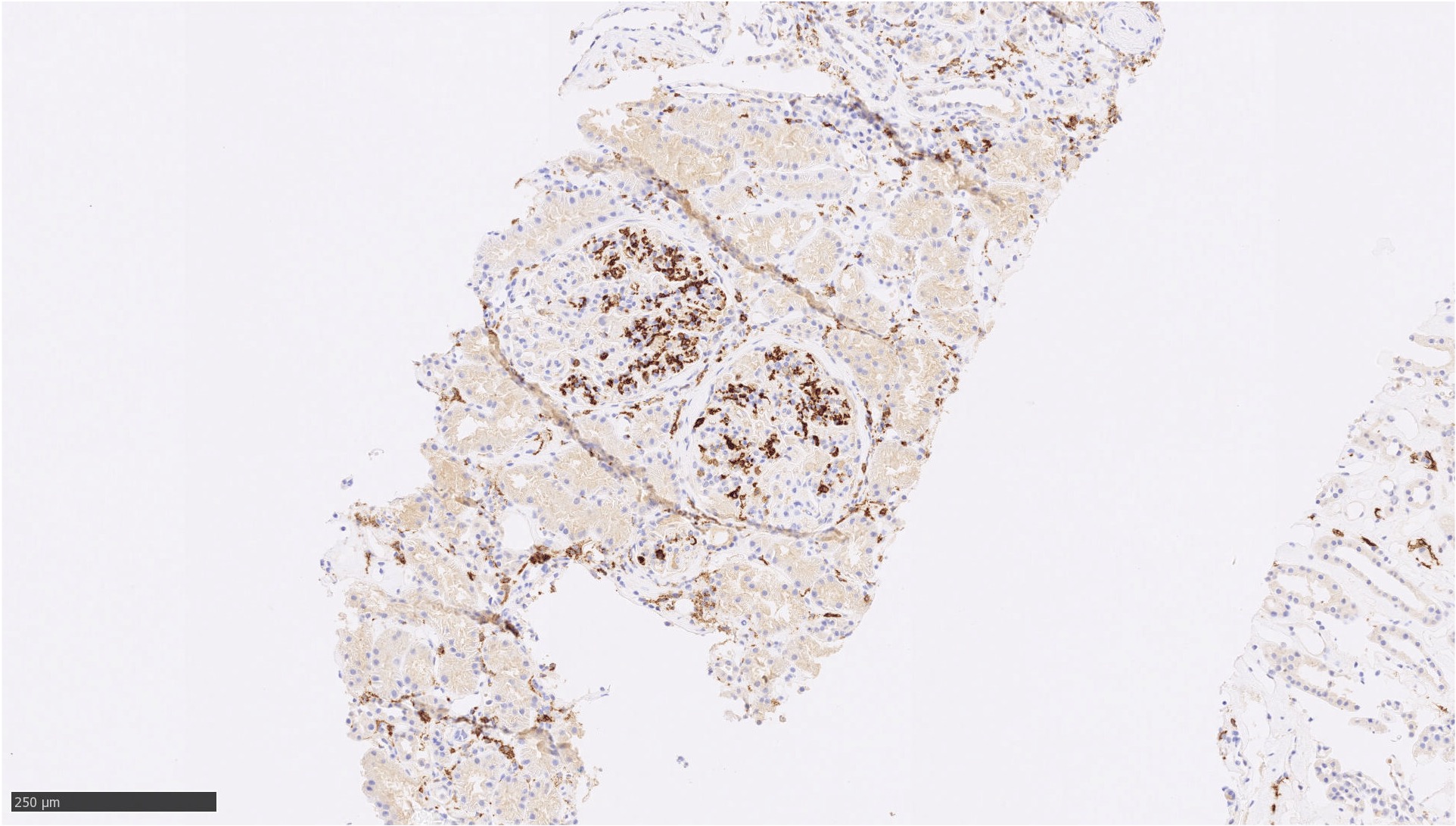

Supplement: Supplementary file 1 — Supplementary Material 1 [file 12882_2025_3986_MOESM1_ESM.jpg]

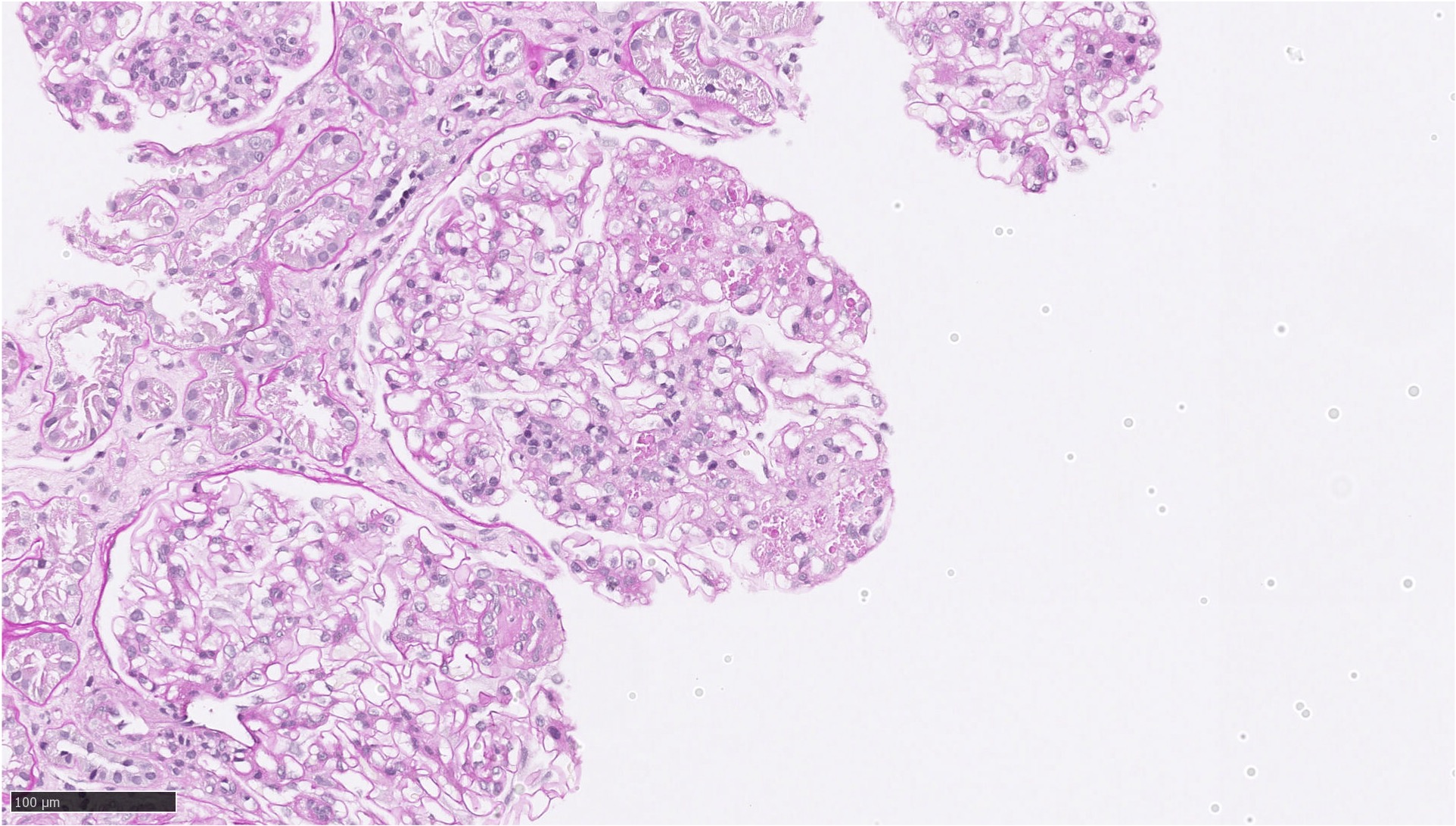

Supplement: Supplementary file 2 — Supplementary Material 2 [file 12882_2025_3986_MOESM2_ESM.jpg]

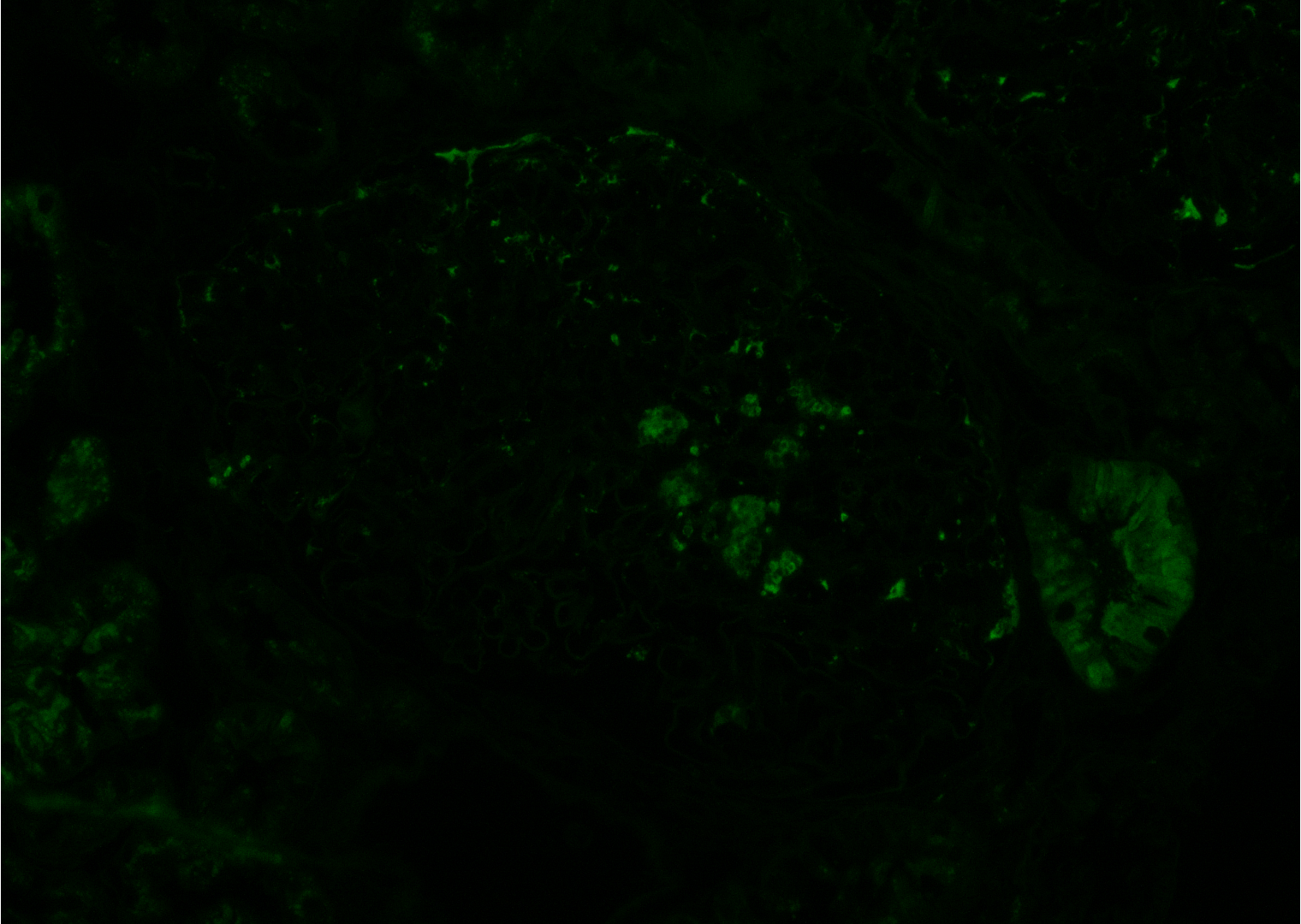

Supplement: Supplementary file 3 — Supplementary Material 3 [file 12882_2025_3986_MOESM3_ESM.png]

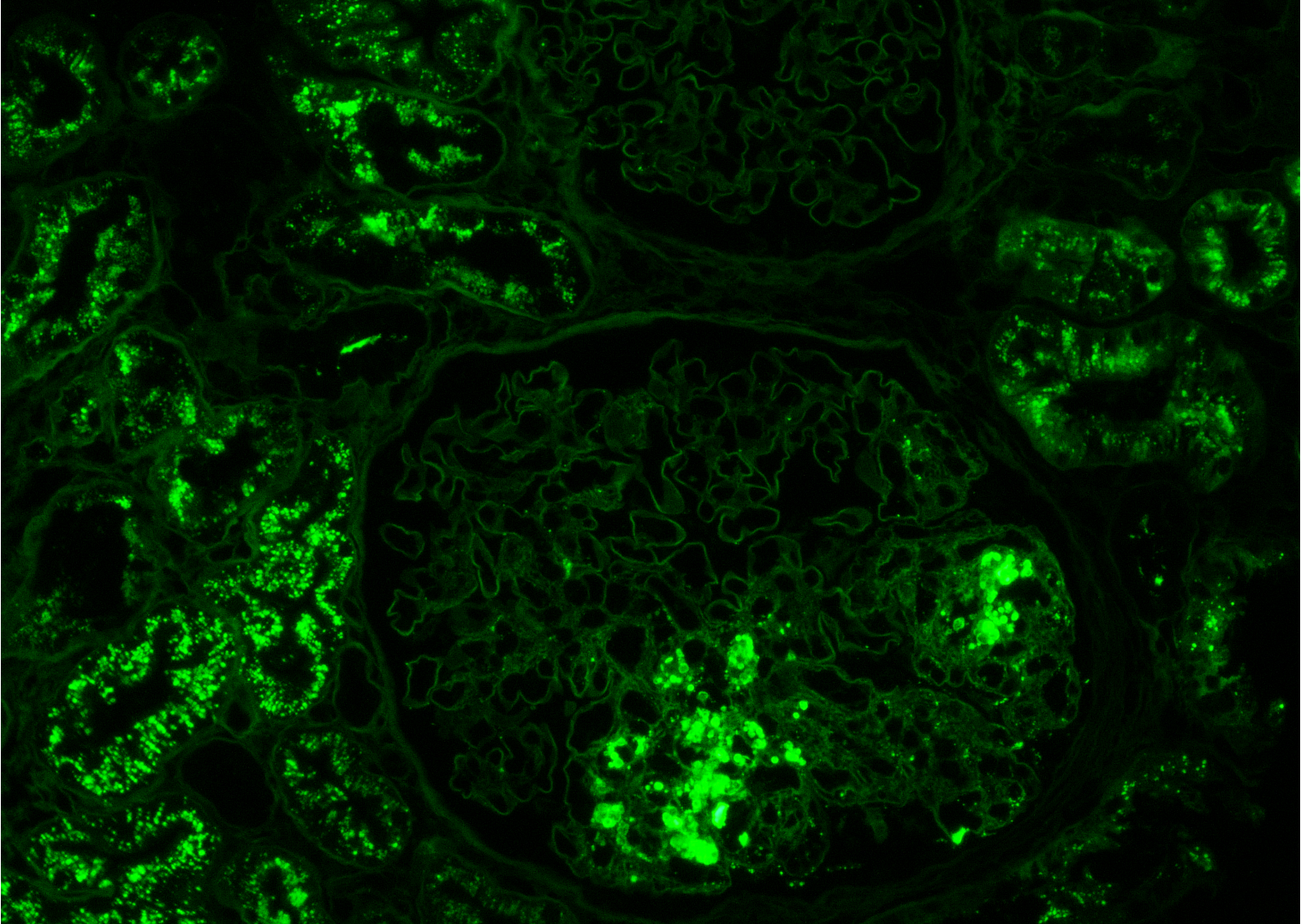

Supplement: Supplementary file 4 — Supplementary Material 4 [file 12882_2025_3986_MOESM4_ESM.png]

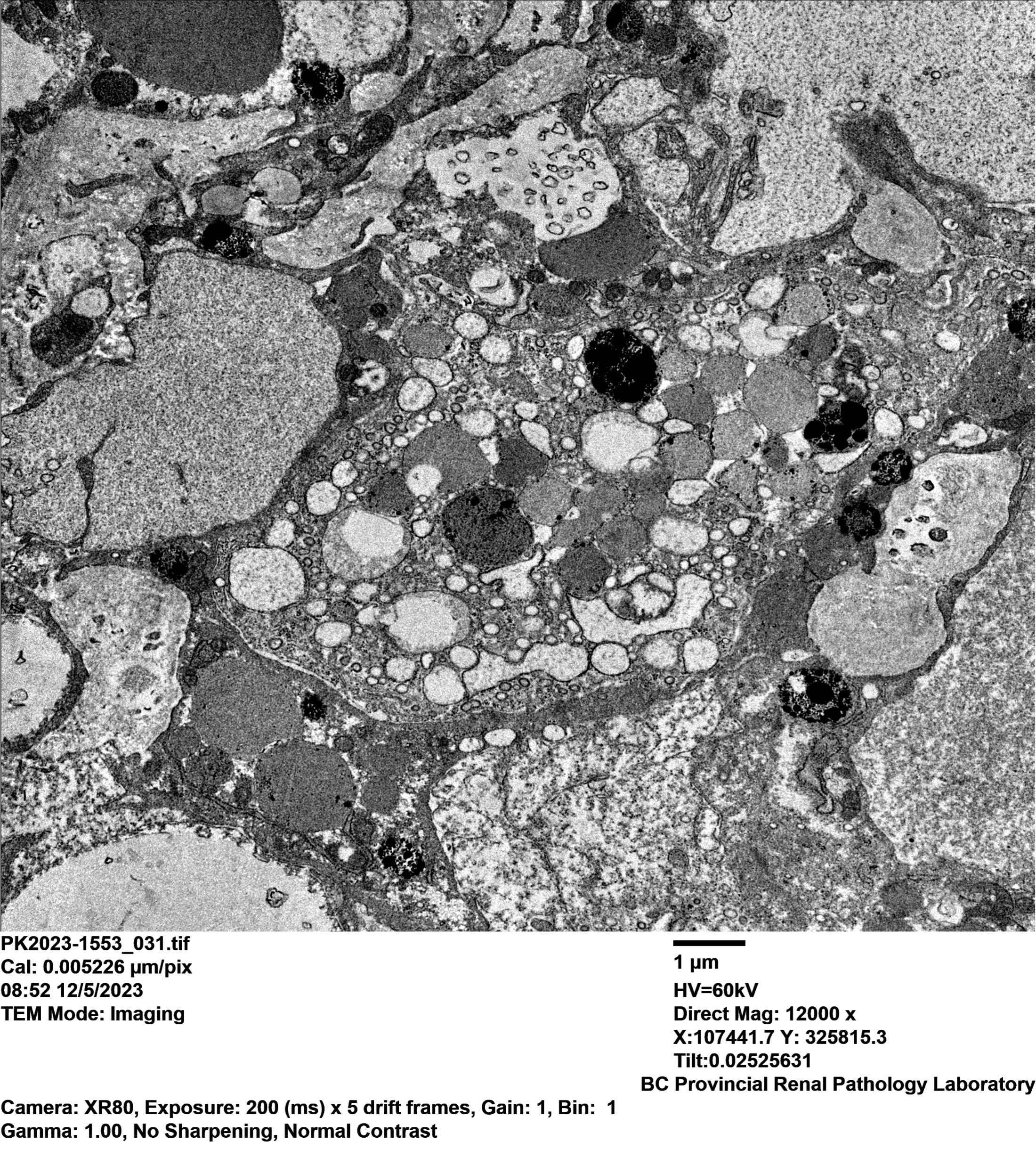

Supplement: Supplementary file 5 — Supplementary Material 5 [file 12882_2025_3986_MOESM5_ESM.jpg]

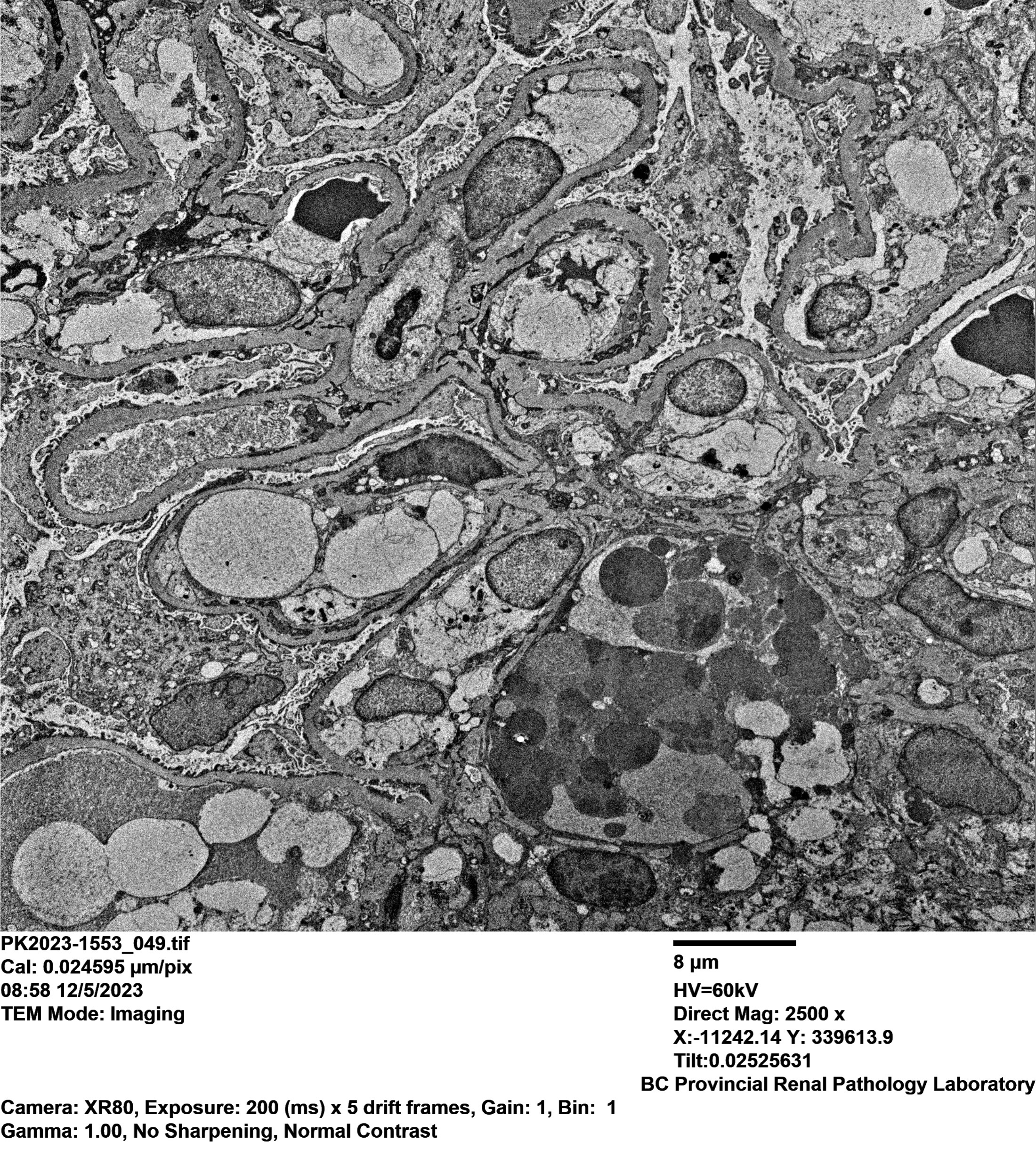

Supplement: Supplementary file 6 — Supplementary Material 6 [file 12882_2025_3986_MOESM6_ESM.jpg]
